# Complete genome reveals genetic repertoire and potential metabolic strategies involved in lignin degradation by environmental ligninolytic *Klebsiella variicola* P1CD1

**DOI:** 10.1371/journal.pone.0243739

**Published:** 2020-12-22

**Authors:** Amanda Oliveira dos Santos Melo-Nascimento, Brena Mota Moitinho Sant´Anna, Carolyne Caetano Gonçalves, Giovanna Santos, Eliane Noronha, Nádia Parachin, Milton Ricardo de Abreu Roque, Thiago Bruce

**Affiliations:** 1 Departamento de Microbiologia, Instituto de Biologia, Grupo de Biotecnologia Ambiental, Universidade Federal da Bahia, Salvador, Brazil; 2 Instituto de Ciências da Saúde, Laboratório de Bioprospecção, Universidade Federal da Bahia, Salvador, Brazil; 3 Departamento de Biologia Celular, Instituto de Biologia, Laboratório de Engenharia de Biocatalizadores, Universidade de Brasília, Brasília, Brazil; 4 Departamento de Biologia Celular, Instituto de Biologia, Universidade de Brasília, Brasília, Brazil; Babasaheb Bhimrao Ambedkar University, INDIA

## Abstract

Lignin is a recalcitrant macromolecule formed by three alcohols (monolignols) predominantly connected by β-aryl ether linkages and is one of the most abundant organic macromolecules in the biosphere. However, the role played by environmental bacteria in lignin degradation is still not entirely understood. In this study, we identified an environmental *Klebsiella* strain isolated from sediment collected from an altitudinal region in a unique Brazilian biome called Caatinga. This organism can also grow in the presence of kraft lignin as a sole source of carbon and aromatic compounds. We performed whole-genome sequencing and conducted an extensive genome-based metabolic reconstruction to reveal the potential mechanisms used by the bacterium *Klebsiella variicola* P1CD1 for lignin utilization as a carbon source. We identified 262 genes associated with lignin-modifying enzymes (LMEs) and lignin-degrading auxiliary enzymes (LDAs) required for lignin and aromatic compound degradation. The presence of one DyP (Dye-decolorizing Peroxidase) gene suggests the ability of P1CD1 strain to access phenolic and nonphenolic structures of lignin molecules, resulting in the production of catechol and protocatechuate (via vanillin or syringate) along the peripheral pathways of lignin degradation. *K*. *variicola* P1CD1 uses aldehyde-alcohol dehydrogenase to perform direct conversion of vanillin to protocatechol. The upper funneling pathways are linked to the central pathways of the protocatechuate/catechol catabolic branches via β-ketoadipate pathways, connecting the more abundant catabolized aromatic compounds with essential cellular functions, such as energy cellular and biomass production (i.e., via acetyl-CoA formation). The combination of phenotypic and genomic approaches revealed the potential dissimilatory and assimilatory ability of *K*. *variicola* P1CD1 to perform base-catalyzed lignin degradation, acting on high- and low-molecular-weight lignin fragments. These findings will be relevant for developing metabolic models to predict the ligninolytic mechanism used by environmental bacteria and shedding light on the flux of carbon in the soil.

## 1. Introduction

Microorganisms are essential in the cycling of a wide range of molecules in the environment, playing critical roles in biogeochemical cycles, and are essential in promoting the balance of life. Among the main activities are those associated with environmental organic carbon cycling. Lignocellulosic biomass represents the most abundant organic compound of vegetal origin on the planet. Therefore, the degradation of lignocellulosic biomass in the environment represents one of the main routes of cycling organic carbon in terrestrial ecosystems. Lignocellulose is a polymeric structure that makes up more than 90% of the plant biomass’s dry weight, and lignin represents approximately 10% to 30% of that amount [1].

Lignin is an amorphous polymeric macromolecule composed of three-dimensional networks of three distinct units of interconnected phenylpropane (p-coumaryl alcohol, coniferyl alcohol, and sinapyl alcohol). The possible arrangements among these units generate a diversity of polymeric structures and vary according to different plant species, resulting in distinct stiffness levels in the plant structure [2]. This diverse and complex panel of molecular arrangements results in structures resistant to various microbial enzymes, causing their environmental recalcitrance. Thus, the lignin degradation process requires a system of enzymes specialized in the attack of specific chemical groups that perform polymer deconstruction in a concerted and ordered way to result in mineralization of the molecule.

Lignin degradation occurs in two stages. The first, called delignification, is the extracellular depolymerization of the primary lignin polymer (the breakdown of the bonds between the precursor alcohols) by the action of lignin-modifying enzymes (LMEs), such as laccases, lignin peroxidase, manganese peroxidase, and dye-decolorizing peroxidase (DyP), and lignin-degrading auxiliary enzymes (LDAs), including aryl alcohol oxidase, glyoxal oxidase, vanillyl alcohol oxidase, cellobiose dehydrogenase, and glucose oxidase, among others, which serve as suppliers of hydrogen peroxide necessary for the activity of LMEs [3]. The second stage refers to the intracellular decomposition of aromatic compounds derived from lignin by enzymes, including oxidases, dehydrogenases, monooxygenases, dioxygenases, and reductases. These enzymes cleave the rings of aromatic compounds and represent groups in the peripheral pathway (where the peripheral aldehydes derived from lignin, in the first stage, are transformed into low-molecular-weight aromatic compounds called intermediary metabolites) and central pathway (where these intermediate are metabolized into substrates that are directed to several metabolic cycles) of aromatic compound metabolism [3, 4]. The metabolization of carbon atoms resulting from the breakdown of the molecular structure of lignin to generate microbial biomass occurs through catalase, thiol peroxidase, glutathione peroxidase, glutathione reductase, and ferredoxins, among others.

Lignin degradation systems were initially well established for fungi such as white-rot fungi. These systems are recognized to completely degrade the lignin molecule through the action of laccase, manganese peroxidase, and lignin peroxidase enzymes. However, interest in bacterial lignin degradation systems has been gaining prominence because of the essential ecological role played by prokaryotes in the environment and their potential biotechnological applications due to characteristics related to the rapid growth, ease of cultivation, and genetic manipulation of bacterial cells [5]. Among the ligninolytic bacteria described in the literature, most of the ligninolytic bacteria described so far belong to the phyla Actinobacteria and Proteobacteria [6].

Currently, the actinobacterium *Rhodococcus jostii* is one of the most relevant bacterial models for lignin degradation [7]. The genomic data from *R*. *jostii* RHA1 revealed the presence of genes involved in the catabolism of aromatic compounds such as biphenyl and alkylbenzene, revealing catabolic pathways and their regulation. Moreover, among *Klebsiella* strains, sequencing data also revealed the presence of Dyp genes and β-ketoadipate [8]. Evidence across the prokaryotes indicates that a common catabolic node for aromatic breakdown is the formation of catechol or protocatechuate, resulting in aromatic ring fission and enzymatic conversion to acetyl-CoA and other constituents of the tricarboxylic acid (TCA) cycle [9, 39, 87]. The genetic bases associated with lignin breakdown are still not well understood, especially in bacteria. Studies focused on the association between genetic variance and ligninolytic potential remain scarce, justifying the necessity of sequencing and establishing ligninolytic microbial models and deepening the studies of already sequenced genomes available in databases.

In this study, we combined phenotypic and genomic characterization of the ligninolytic strain *K*. *variicola* P1CD1 isolated from sediment samples collected in an altitudinal region of the Caatinga biome, a unique Brazilian biome whose borders are strictly within the national territory, whose microbial genetic repertoire is still poorly characterized. We identified the genetic repertoire that composed its lignin degradation system and performed an extensive genome-based metabolic reconstruction, establishing the potential routes associated with the metabolic strategies used by *K*. *variicola* P1CD1 for lignin deconstruction. The present findings have relevance in microbial molecular ecology, shedding light on the strategies used by ligninolytic prokaryotes in converting environmental lignocellulosic biomass.

## 2. Material and methods

### 2.1. *K*. *variicola* P1CDI strain isolation

The sediment was collected in the dry season (February 2015) along the Morro do Chapéu trail in the Chapada Diamantina National Park (227649, 8568822 UTM). The temperature in this region was approximately 28 °C, and the humidity ranged from 60 to 98%. Samples consisted of 30 g of soil sediments present along the cracks on the rock floor located at the Morro do Chapéu plateau (1,700 m above sea level). After the removal of surface litter, the sediment was collected at a depth of 3 cm. The material was stored in sterile plastic bags and kept on ice until used for experiments. The isolation of microbes was driven using minimal media containing lignin as the only carbon source. Minimal medium was supplemented with alkali lignin (MML) (0.12% NaNO3, 0.3% KH2PO4, 0.6% KH2PO4·2H2O, 0.2% MgSO4·7H2O, 0.005% CaCl2·2H2O, 0.0001% ZnSO4·7H 2O, 0.001% MnSO4·7H2O, and 1% alkali lignin) [10]. Five grams of sediment was inoculated in 50 mL of MML and diluted to 10 −11, and 100 μl was directly spread on solid MML (10% agar). The plates were incubated at 30°C for 48 h. P1CD1 single colonies were recovered and replated on another solid MML plate (2 times) before being stored in MML supplemented with 20% glycerol at -80 °C. Moreover, the strains were reactivated from frozen stocks in liquid LB medium. After 24 h, the bacteria were inoculated on solid MMKL (kraft lignin) and incubated at 30°C.

### 2.2. Ligninolytic phenotype

#### 2.2.1. Growth in the presence of kraft lignin

Lignin was recovered from the industrial black liquor, the residue of the LignoBoost^®^ process. The black liquor was composed of the hybrid eucalyptus *E*. *Grandis x E*. *Urophylla*, a hardwood tree.

The strain *K*. *variicola* P1CD1 grew in the presence of kraft lignin. The minimal medium with kraft lignin (MMKL) (0.12% NaNO3, 0.3% KH2PO4, 0.6% KH2PO4·2H2O, 0.2% MgSO4·7H2O, 0.005% CaCl2·2H2O, 0.0001% ZnSO4·7H 2O, 0.001% MnSO4·7H2O, and 1% kraft lignin) was supplemented with 20 nmol −1 of glycerol. Cultures were incubated in a rotary shaker at 200 rpm and 30°C for five days. All experiments were performed in triplicates. The bacterial growth curve was determined by 620_OD_ measurements every 24 hours.

#### 2.2.2. Growth in the presence of aromatic dyes

Growth in the presence of aromatic dyes as the sole carbon source on solid medium was evaluated as described by Bandounas et al. (2011) [11] using a solid minimal medium (MM) containing 25 mg l −1 MB, 25 mg l −1 toluidine blue (TB) and methylene blue (MB), supplemented with 40 nmol −1 glycerol. The isolated colony was streaked on the plates and incubated at 30 °C for 72 h.

The pre inoculum for dye degradation in the liquid medium assay was prepared by inoculating isolated colonies in 50 mL of minimal medium containing 25 mg l −1 MB or 25 mg l −1 TB and MB, supplemented with 40 nmol −1 glycerol, and incubated at 30 °C for 24 h. The culture was centrifuged for 5 minutes at 4000 rpm, the supernatant was removed, and the cell pellet was recovered with 1 mL of fresh MM with the dyes and glycerol. Then, 20 μL was inoculated in 180 μL of MM with the dyes and glycerol and incubated at 30 °C for 72 h. The absorbance variation was monitored using a wavelength of 620 nm every 1 hour.

#### 2.2.3. Residual lignin dry weight

The relative reduction in Kraft lignin content was measured in comparison to the uninoculated sample using a variation of the classical Klasson method applied in previous studies with focus on microbial ligninolytic characterization [12, 13]. In order to estimate relative Kraft lignin consumption by bacteria, samples were acidified with 1M HCl to pH 1–2. Then, samples were centrifuged for 10 min at 5000 rpm to obtain precipitated lignin. After centrifugation, the supernatant was removed and precipitated lignin was washed and neutralized with deionized water. After that, placed into membrane filters and dried for 24h at 55°C oven. Membranes were then weighted to evaluate relative Kraft lignin degradation after five days of growth.

### 2.3. Genome sequencing, assembly, and annotation

#### 2.3.1. Genome sequencing

Genomic DNA extraction was performed using the commercial kit PureLink Genomic DNA Mini Kit (Invitrogen) according to the protocol and confirmed after electrophoresis using a Nanodrop spectrophotometer. The sample concentration was 100 ng/μL, and a 260/280 nm > 1.8 ratio of purity was used for random insert library construction. DNA was mechanically sheared, and fragments with a size range of 150–300 bp were used for the library preparation, which was performed using the NEBNext Ultra DNA Library Prep Kit (Illumina). DNA inserts were sequenced on an Illumina HiSeq4000 system using the Illumina HiSeq 3000/4000 SBS Kit for paired-end (PE) sequence generation. The generated sequences were checked for quality and integrity, analyzing the raw data through MD5Checker version 3.3 (http://getmd5checker.com/).

#### 2.3.2. Genome assembly

The read quality assessment was performed using the FastQC tool (http://www.bioinformatics.babraham.ac.uk/projects/fastqc), and *de novo* genome assembly was performed through the SPAdes Genome Assembler (version 3.9) [14]). In SPAdes, we used the parameter "cov-cutoff auto" to complete the *de novo* assembly with Kmer 127, generating the best result, obtaining 45 contigs with 5,615,206 bp, which were organized into scaffolds using *K*. *variicola* At-22 (GenBank: NC013805.1) as a reference strain.

The scaffolds were organized and oriented using CONTIGuator 2.3 (http://contiguator.sourceforge.net/) [15]. The CONTIGuator excludes duplicate contigs, small contigs, and inadequate coverage contigs. The 45 contigs were used to build 15 scaffolds (30 contigs were excluded in this step). The initially excluded contigs were blasted against the NCBI and UniProtKB/Swiss-Prot databases to confirm functional information (homology) associated with the closest phylogenetic neighbors. We found a total of 14 remaining gaps. The gap-filling process was performed in three stages, based on (i) CLC Genomics Workbench 7.0 (CLC-gw) (Qiagen, USA), (ii) GapBlaster to solve repetitive and overlapping gap regions between contigs, and (iii) manual curation.

In the first stage, the reads were mapped along the organized scaffolds using the default parameters in CLC Genomics Workbench 7.0 (CLC-gw). The overlapping of *K*. *variicola* P1CD1 reads in the terminal regions that precede the gaps between the scaffold was oriented by the genome reference (*K*. *variicola* At-22) towards the internal regions, enabling the gap-filling step. A total of 8 RNA gaps were filled. In the second stage, the gaps were filled using the GapBlaster software [16] for alignment and inclusion of contigs excluded by CONTIGuator 2.3 (due to small length, low coverage, and duplicity with genome reference region) in the scaffolds. We used GapBlaster to fill four gaps by aligning the 30 excluded contigs against the resulting scaffold from the CLC-gw gap filling. Finally, the two remaining gaps were manually curated to solve overlaps between the contig using Artemis 16.0.0 software [17] and Blast analysis [18].

In the end, we obtained the complete genome in a single contig, which was deposited in NCBI under accession number CP033631.

#### 2.3.3. Genome annotation and metabolic pathway reconstruction of the lignin degradation system in *K*. *variicola* P1CD1

*2*.*3*.*3*.*1*. *Annotation of genetic repertoire involved with aromatic compound degradation*. The genes were annotated on the Rapid Annotation using Subsystem Technology (RAST) server [19]. Initially, the open reading frames present in the metabolism of the aromatic compound category and the lignin fragment degradation subsystem were inspected. However, we also found genes involved in lignin degradation outside of those metabolic compartments. Thus, we performed inspection using the Subsystem Browser tool based on the gene roles to identify other coding genes involved in lignin degradation outside of the metabolism of aromatic compound category or lignin fragment degradation subsystems. We opted for that search because there is still an inherent database bias associated with the poorly established bacterial lignin degradation metabolism. Misannotation may occur for genes already assigned to one metabolism but not flagged for another. Moreover, based on the literature, we included the genes absent in subsystems but with activities involved in the two stages of lignin modification by searching the role and/or EC number. All the genes involved in aromatic compound degradation found in the complete genome of *K*. *variicola* P1CD1 are presented in S1 Table. The circular map was built using the program BLAST Ring Image Generator (BRIG) [20], highlighting the location of genes involved in lignin degradation.

*2*.*3*.*3*.*2*. *Genome-based metabolic pathway reconstruction of lignin degradation metabolism*. We found a total of 94 functional roles distributed in 12 curated subsystems harboring essential metabolic pathways for aromatic compound degradation. On the other hand, the coding gene-based search for the dehydrogenase, oxidase, reductase, monooxygenase, dioxygenase, transporter, transcriptional regulatory, hydroxylase, peroxidase, transferase, lyase, hydrolase, isomerase, ligase, superoxide, and redox activities retrieved 168 genes (functional roles) not associated with the subsystems or categories pre-established by RAST server annotation.

The metabolic network representation was separated according to the type of modification performed by the target activity. The first stage is represented by the enzymatic attack of the lignin molecule to generate monolignols and smaller phenolic fragments, and the second stage is represented by the deconstruction of the ring structures to generate intermediates through the peripheral and central pathway. The subsystems constituting the metabolism of the aromatic compounds were the starting point for assembling the complete genetic repertoire and reconstructing the metabolic pathways used by *K*. *variicola* P1CD1. Reconstruction of the degradation metabolism was performed by KEGG (Kyoto Encyclopedia of Genes and Genomes) annotation via the RAST server. As expected, the aromatic compound degradation metabolism via RAST was initially identified as incomplete in the P1CD1 genome. The first stage was established based on the search for genes coding for specific activities involved in phenolic and nonphenolic structures well established in the literature [3, 19]. The second stage was set by manual identification of the activities missing in the metabolic routes but available in the established genetic repertoire (including genes present and absent in the subsystems). The search was based on EC numbers representing each of the pathways present in the routes for benzene degradation, 4-hydroxybenzoate degradation, aminobenzoate degradation, phenylalanine metabolism, and tyrosine metabolism.

### 2.4. Genomic taxonomy

The taxonomic assignment of the P1CD1 strain was performed in two stages: i) using a polyphasic molecular approach throughout the MLSA (multilocus sequence analysis) by the alignment of conserved genes and ii) *in silico* DNA-DNA hybridization and average nucleotide identity by whole-genome alignment.

#### 2.4.1. MLSA and phylogenetic reconstruction

The P1CD1 strain was initially identified as a member of the *Klebsiella* genus by using the Basic Local Alignment Search Tool (BLAST) against rRNA 16S gene sequences filtered from the genome based on RAST annotation, as described in the following section addressing genome annotation. Based on the results, we searched for the type strains of species belonging to the *Klebsiella* genus with the complete genome, according to the official prokaryotic standing nomenclature available in EUZEBY (http://www.bacterio.net/). The most similar strains with complete genome sequences available in NCBI were added to the phylogenetic reconstruction. We found only three type strains with complete genome sequences available in the NCBI public database (*K*. *pneumoniae* ATCC BAA-2146, *K*. *quasipneumomniae* ATCC 700603, and one *K*. *variicola* DSM 15968). The other non-type closest phylogenetic neighbors used in the MLSA were determined based on the P1CD1 rRNA 16S gene similarity against NCBI and the Ribosomal Database Project (RDP) databases. Reference genomes of members belonging to the *Enterobacteriaceae* family were used as outgroups. We selected a total of 26 strains to build the *K*. *variicola* P1CD1 MLSA. Among them, we found three type strain complete genomes, seven complete reference genomes, fifteen most similar complete genomes, and one scaffold genome sequence.

The conserved genes were selected using the AMPHORANet software [21]. Only genes present in all strains were used. We concatenated a total of 32 genes for each isolate and performed multi alignment (*dnaG*, *frr*, *infC*, *nusA*, *pgk*, *pyrG*, *HX2*, *rplA*, *rplB*, *rplC*, *rplD*, *rplE*, *rplF*, *rplK*, *rplL*, *rplM*, *rplN*, *rplP*, *rplS*, *rplT*, *rpmA*, *rpoB*, *rpsB*, *rpsC*, *rpsE*, *rpsI*, *rpsJ*, *rpsK*, *rpsM*, *rpsS*, *smpB* and *tsf)*. The genes were first manually concatenated and aligned with MEGA X software using the Muscle algorithm and manually checked [22]. There was a total of 7688 positions in the final dataset. The evolutionary history was inferred using the maximum likelihood method based on a JTT model and bootstrap test (500 times). The analysis involved 27 amino acid sequences. All positions with less than 95% site coverage were eliminated. That is, fewer than 5% alignment gaps, missing data, and ambiguous bases were allowed at any position.

#### 2.4.2. *In silico* DNA-DNA hybridization and ANI (average nucleotide identity)

Taxonomic classification was confirmed based on *in silico* DDH (DNA-DNA hybridization) and ANI analysis to complete the taxonomic assignment. The *in silico* DDH was performed using the Genome to Genome Distance Calculator (GGDC) (https://ggdc.dsmz.de/home.php) [23], and the ANI was determined using the JSpeciesWS Online Service (http://jspecies.ribohost.com/jspeciesws/) [24]. In both analyses, the genome of P1CD1 was aligned against the genomes of *Klebsiella variicola* At-22, *Klebsiella variicola* DSM 15968, and *Klebsiella variicola* 342. We considered values > 80% and > 95% similarity for DDH and ANI, respectively, as belonging to the same species. Based on the agreement between the DDH and ANI results, we confirmed the taxonomic classification of the P1CD1 strain.

## 3. Results

### 3.1. The ligninolytic potential of *K*. *variicola* P1CD1

*K*. *variicola* P1CD1 was isolated in the presence of alkali lignin as the sole carbon source and in the presence of kraft lignin in solid and liquid media. The growth curve revealed a 24 hours log phase and reached the higher biomass formation between 12 and 96 hours (S1 Fig).

**Fig 1:**
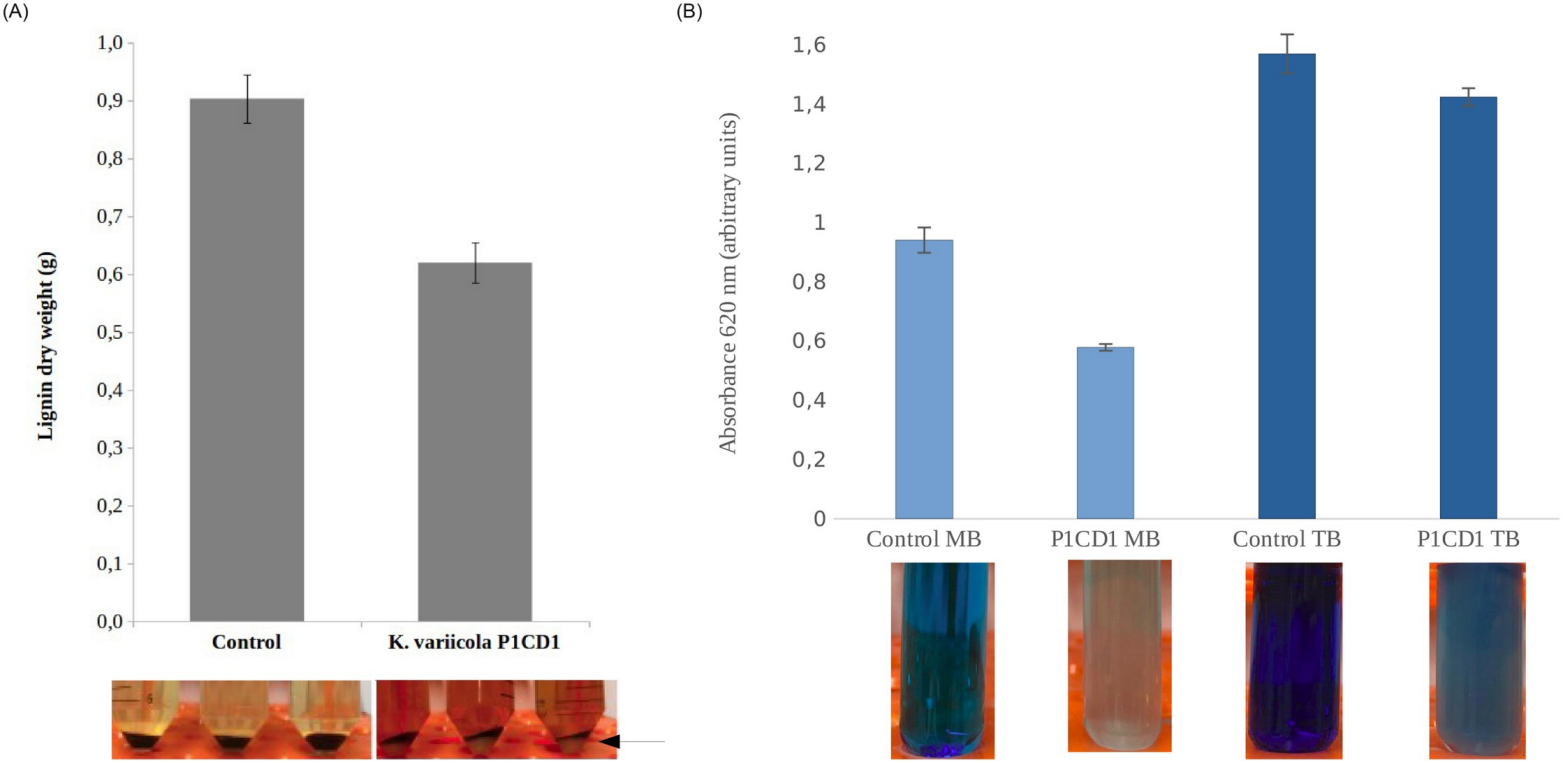
The phenotype-based ligninolytic potential of *Klebsiella variicola* P1CD1. A) The semi-quantitative approach to demonstrate the conversion of non-soluble lignin by *K*. *variicola* P1CD1. Dry weighted residual lignin was reduced in 30% in the presence of *K*. *Variicola* P1CD1 after 96 hours and is possible to observe the biomass indicated by the black arrow (not present in control samples), indicating the conversion of lignin in microbial biomass. B) Decolouration of aromatic dyes (methylene blue and toluidine blue) in 24 hours indicating the ability to breakdown aromatic lignin fragments.

A semi-quantitative approach was used to detect the conversion of kraft lignin in microbial biomas. we weighed the residual lignin in the absence and presence of the K. variicola P1CD1 strain after 96 hours of growth. It is possible to observe a decrease in insoluble lignin and an increase in microbial biomass in the fraction referring to the precipitate recovered by centrifugation (Fig 1A). We observed the amount of residual non soluble lignin fraction was 30% higher in negative control (culture media with no inoculation) in comparison with the *K*. *variicolla* P1CD1 samples. Moreover, aiming to detect potential ability to convert lignin fragments, we performed the microbial growth in the presence of aromatic dyes and we found positive results for methylene blue and toluidine blue (no grwoth was observed for malchita green and congo red. Fig 1B. The dye degradation assay revealed decreases of 43% and 32% in the absorbance of the culture in the presence of toluidine blue and methylene blue, respectively in liquid media.

### 3.2. Genome-based taxonomy classification of *K*. *variicola* P1CD1

**Fig 2:**
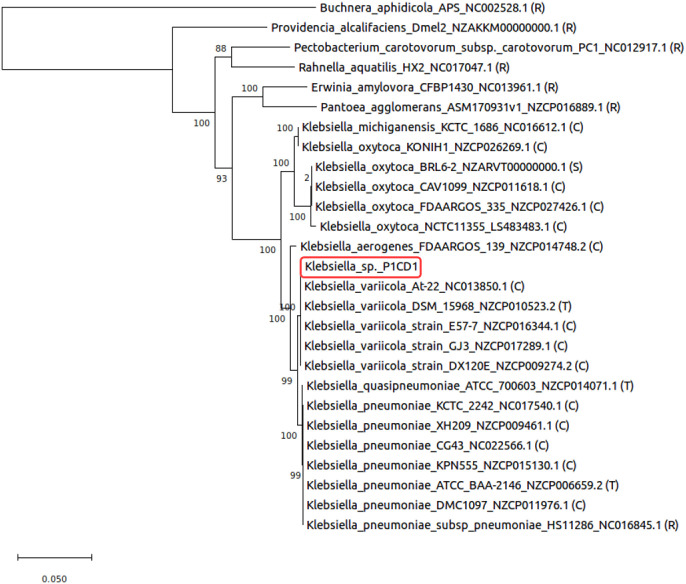
Phylogeny of *Klebsiella variicola* P1CD1 (red box). The genes were manually concatenated and aligned by MEGA X software using the Muscle algorithm and manually checked. The evolutionary history was inferred using the maximum likelihood method based on a JTT model and bootstrap test (500 times). R—Reference genome (complete, non-type strain); C—Complete genome (not reference, non-type strain); T—Type strain (complete genome); S—Scaffolds.

The phylogeny resulted in three distinct clusters limited by the closest *Klebsiella* type strains presenting whole sequenced genomes in the database (*K*. *variicola*, *K*. *oxytoca*, and *K*. *pneumoniae*). The P1CD1 strain is allocated in the cluster associated with the type strain *K*. *variicola* DSM15968 (Fig 2). Genome-based taxonomy identified 94.50% and 98.92% similarity for DDH and ANI, respectively, when comparing the *K*. *variicola* P1CD1 genome against that of the type strain *K*. *variicola* DSM15968. Since the same species, cut-off values are > 70% and > 95% for DDH and ANI respectively, the values confirm that the strains belong to the same species, indicating that P1CD1 is a new strain of *K*. *variicola*. However, the non-type strain *K*. *variicola* At-22 genome showed the highest similarity to the *K*. *variicola* P1CD1 genome from DDH and ANI analysis (95% and 99%, respectively).

### 3.3. Genomic functional profile of ligninolytic *K*. *variicola* P1CD1

#### 3.3.1. General features

Genomic sequencing generated a total of 6,795,156 sequences with an average length of 150 bp, providing nearly 180-fold genome coverage (S2 Fig). The complete genome has a total length of 5,633,647 bp, with a GC content of 57.3%, 86 tRNAs and 22 rRNAs (7 operons and 1 5S) and 5,451 CDS (coding DNA sequences). Among the coding DNA sequences (CDS), 4,210 are predicted proteins and 1,241 are hypothetical proteins, but only 2,725 (49.99%) are assigned, along with the 25 subsystem categories assigned through the Rapid Annotation Subsystems Technology (RAST). The complete list of subsystems and gene counting for the genome of *K*. *variicola* P1CD1 is shown in S3 Fig. The majority of sequences were assigned under the carbohydrate subsystem category (513 sequences), followed by amino acids and derivatives (486 sequences), representing 36.66% of all coding genes assigned to subsystems. The metabolism of aromatic compounds presented 83 sequences (2.57% of the genes), appearing as the eleventh most abundant subsystem in the genome.

The metabolism of aromatic compounds harbors subsystems related to lignin-derived aromatic compound degradation. The metabolic pathways for the degradation of aromatic compounds derived from lignin harbor a total of 12 subsystems (quinate degradation, benzoate degradation, 4-hydroxybenzoate degradation, hydroxyaromatic decarboxylase family, catechol pathway, protocatechuate pathway, 4-hydroxyphenylacetic acid catabolic pathway, central meta-cleavage pathway, salicylate, and gentisate catabolism, gentisate degradation, aromatic amine catabolism, and lignin degradation fragments) in *K*. *variicola* P1CD1. We found an apparent contradiction among genotype and phenotype. Even when growing in lignin as the sole carbon source, some of the subsystems in the genome of *K*. *variicola* P1CD1 were incomplete. Based on that result, we searched among the coding genes outside the metabolism of aromatic compounds for activities strictly related to lignin degradation to establish the complete genetic repertoire of genes potentially involved in the lignin-degrading system of the P1CD1 strain.

#### 3.3.2. The genetic repertoire of *K*. *variicola* P1CD1 for lignin and aromatic compound degradation metabolism

The genetic repertoire of *K*. *variicola* P1CD1 for lignin and aromatic compound degradation presented a total of 262 genes potentially involved in these processes (considering the genes present and absent in the subsystems) (S4–S6 Figs and S1 Table). Among the activities involved in the first stage of lignin degradation of lignin and aromatic compounds, the dehydrogenases showed the highest contribution (46 genes), followed by transferases (44 genes) and lyases (35 genes). On the other hand, the genes involved in activities performed in the second stage of lignin and aromatic compound degradation were identified as monooxygenases (17 genes), reductases (18 genes), hydrolases (16 genes), peroxidases (13 genes), dioxygenases (11 genes), isomerases (9 genes) and oxidases (7 genes) (S2 Table).

The genetic repertoire was used to populate the metabolic pathways present in the routes, with 340 previously established for the degradation metabolism of lignin fragments and aromatic compounds and 341 via KEGG. We performed metabolic reconstruction of the pathways in *K*. *variicola* P1CD1. We identified 33 coding sequences harboring 32 genes (two versions of *gstB*_2) distributed among five activities associated with the first stage of lignin degradation (deconstruction of phenolic and nonphenolic structures). On the other hand, we found 32 coding genes among 23 activities associated with the second stage, suggesting the potential ability to degrade aromatic compounds derived from stage one of lignin degradation (a total of 83 genes in the two stages).

#### 3.3.3. Reconstruction of the lignin degradation metabolism of *K*. *variicola* P1CD1

*3*.*3*.*3*.*1*. *Stage one (primary lignin molecule degradation)*. The *K*. *variicola* P1CD1 strain possesses the genes necessary to perform the first stage of lignin degradation, which involves an enzymatic attack that results in the release of lignin fragments arising from cleavage of β-aryl-ether (β-O-4') linkages of phenolic and nonphenolic structures. The repertoire involved with the first stage consists of enzymes not present in the subsystems (S1 and S2 Tables). All the genes were blasted against the UniProt database. Most of them presented identity above 90% compared to most previously identified genes in other deposited sequences of *Klebsiella* strains. The lowest identity was found for one gene identified as a FAD-linked oxidase/vanillyl-alcohol oxidase (AA4) (46.4%) compared to the *Proteus vulgaris* gene. According to the obtained results, *K*.*variicola* P1CD1 possesses the genetic repertoire required to deconstruct phenolic and nonphenolic structures. The *K*. *variicola* P1CD1 repertoire contains genes coding for activities involved in the attack of the lignin polymer by the action of DyP, multicopper phenol oxidases/laccase, and β-ether/glutathione-S-transferases.

**Fig 3:**
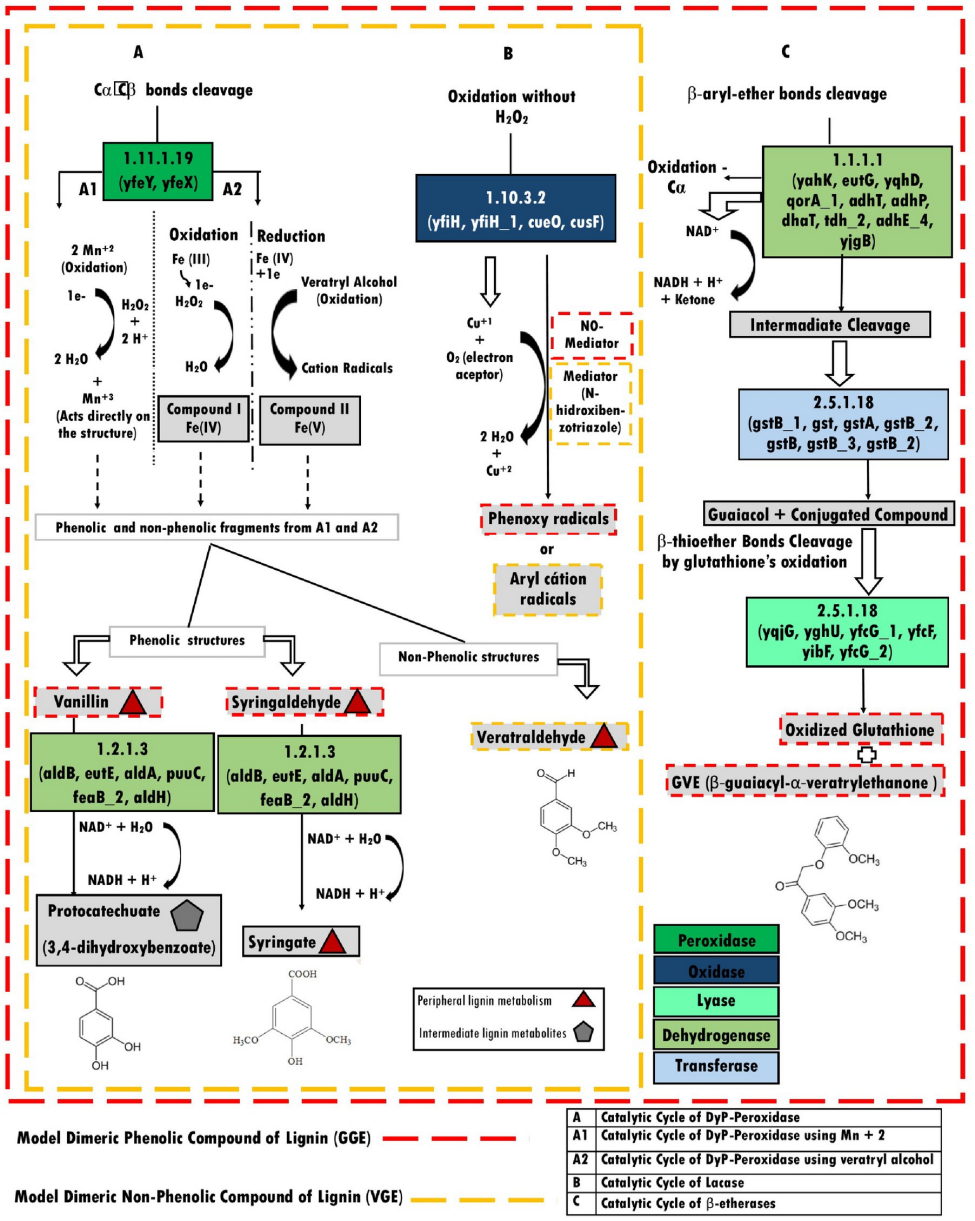
The genome-wide-based metabolic reconstruction of dissimilatory metabolism for phenolic (red line) and nonphenolic (yellow line) lignin structures in *K*. *variicola* P1CD1. A—DyP gene activation with vanillin and syringaldehyde as the final products of peripheral pathways of lignin degradation via aldehyde dehydrogenase activity. A1 –The DyP activity via Mn2+ ion utilization, and A2 –The DyP activity via iron or veratryl alcohol utilization as mediators. B—Laccase activity via Cu1+ and oxygen utilization as an electron acceptor. C– β-etherase bond cleavage via alcohol dehydrogenase for the primary oxidation of glutathione. Red triangles represent peripheral intermediates, the gray pentagon represents the central intermediate of lignin degradation, and the color legend represents the activity associated with its respective metabolic pathway.

*3*.*3*.*3*.*1*.*1*. *Common strategies for the degradation of phenolic and nonphenolic structures*. Based on the classical pathways for phenolic structure degradation, we identified three catalytic cycles by which the phenolic structures from lignin are converted into smaller molecules by the *K*. *variicola* P1CD1 strain (Fig 3). We identified two strategies shared with phenolic and nonphenolic structures, and one used only to deconstruct the phenolic structures.

The catalytic cycle of the DyP is characterized by oxidative cleavage of the phenolic Cα-Cβ bond by enzymes encoded by the genes *YfeY* (encapsulating protein for DyP) and *YefX* (DyP—*YfeX*-like subgroup) (S1 and S2 Tables).

Based on the *K*. *variicola* P1CD1 metabolic reconstruction, the initial steps of aromatic compounds degradation via oxidative cleavage of the phenolic Cα-Cβ bond is performed by Dyp activities. DyPs are heme peroxidase proteins equivalent to the fungal oxidases in lignin degradation. We identified the gene *YefX* (DyP—*YfeX*-like subgroup) among the *K*. *variicola* P1CD1 ligninolytic genetic repertoire. The Blast analysis revealed the *K*. *variicola* P1CD1 YefX present 90% of homology with the *YefX* sequence of *K*. *pneumoniae* IS39 and 90% with the *YefX* from *E*.*coli* O157:H7 (UniProt Id: W1I4T7 and Q8XBI9, respectively). Moreover, *K*. *variicola* P1CD1 *YefX* presented 93% of similarity with the gene TatDyPrx of *Trichoderma atroviride* (Peroxidatabase Id: 10123), 88.9% of similarity with a putative *DypB* gene of *Enterobacter lignolyticus* SCF1 (Protein database—PDB Id: 5VJ10), and 35% of similarity with *DypB* of *Rodhococcus jostii* RHA1 (Protein database—PDB Id: 3VEE). We also found the gene *XfeY* associated with the initial steps of phenolic compounds in the reconstructed metabolic model. The *K*.*variicola* P1CD1 *XfeY* gene presented 97% of similarity with *XfeY* of *Klebsiella pneumoniae* IS43 (UniProt Id: A0A0H3GTD5) and 75% with the *XfeY* of *Escherichia coli* K12 (UniProt Id; P76537). We found the gene *YfeY* closely related to the *YfeX* gene in the *K*. *variicola* P1CD1 genome in RAST annotation. *YefY* is between 1460382–146057 and *YfeX* 1461052–1461951 (S1 Table) in the same genomic context, suggesting they are operonically arranged.

The attack on the phenolic and nonphenolic structures by DyP peroxidases can occur with the oxidation carried out by hydrogen peroxide, with consequent loss of two electrons to form the compound I Mn+2 intermediate to generate Mn+3, which acts directly on the lignin molecule to oxidize the phenolic moiety (Fig 3A, catalytic cycle A1). DyP also has catalytic activity involving the oxidation of veratryl alcohol (VA) by hydrogen peroxide to form radical cations, causing the depolymerization of lignin, or directly via hydrogen peroxide decoupling to promote phenolic structure oxidation (Fig 3, catalytic cycle A2). Both pathways are dependent on hydrogen peroxide, thus generating the peripheral metabolite veratraldehyde as a product of the nonphenolic structure and the peripheral metabolites vanillin and syringaldehyde as products of the phenolic structure. The set of aldehyde-alcohol dehydrogenases (*aldB*, *eutE*, *aldA*, *puuC*, *feaB_2*, *and aldH*) may convert the syringaldehyde into syringate and vanillin protocatechuate, an intermediate metabolite of lignin fragment and substrate for enzymatic activities in the second stage of lignin degradation.

Alternatively, the catalytic cycle of the multicopper phenol oxidase/laccase involves phenolic oxidation mediated by *YfiH* (polyphenol oxidase), *YfiH*_1, and *CueO* (blue copper oxidase) genes, using copper ions and oxygen molecules as the electron acceptor, generating two water molecules and phenoxy radicals in the absence of hydrogen peroxide. We identified four genes associated with Laccase activity in *K*. *variicola* P1CD1 genome (two versions of *YfiH*—Multicopper polyphenol oxidase; *CueO*—Blue copper oxidase CueO precursor and *CuSF*—Copper-binding protein). The gene *YfiH* (Multicopper polyphenol oxidase) showed 97% of similarity with a hypothetical protein (gene r’rh) of *K*. *pneumoniae* ATCC 700721 (UniProt Id: A6TCK1), and *YfiH*_1 (Laccase domain-containing protein) showed 100% with gene *YfiH*_2 (UniProt Id: A0A1W1JYN4) of an uncharacterized *K*. *pneumoniae* and 61% *YfiH* of *K*. *pneumoniae* ATCC 13884 (UniProt Id: A0A378EG74). The gene *CueO* (Blue copper oxidase CueO precursor) showed 100% with Multicopper oxidase CueO of *K*. *variicola* ATCC BAA-830 (UniProt Id: A0A3G5D7T0). The *CusF* (Copper-binding protein) gene showed 97% of similarity with a predicted copper-binding protein of *K*. *pneumoniae* (UniProt Id: A0A378AUY2) and 56% with the curated protein CusF of *E*.*coli* K12.

*3*.*3*.*3*.*1*.*2*. *Particular pathway for the degradation of phenolic structures*. The last pathway identified among the phenolic models is the catalytic cycle of β-ether/glutathione-S-transferase, which performs phenolic Cα oxidation mediated by enzymes encoded by the genes *yahK*, *eutG*, *yqhD*, *qorA_1*, *adhT*, *adhP*, *dhaT*, *tdh_2*, *adhE_4*, *and yjgB* (Fig 3C). The intermediates are cleaved by β-etherase, followed by the addition of glutathione (*gstB_1*, *gst*, *gstA*, *gstB_2*, *gstB*, *gstB_3*, *and gstB_2*) to the Cβ carbon, generating guaiacol and a conjugated glutathione compound that may be modified by other glutathione enzymes (*yqjG*, *yghU*, *yfcG_1*, *yfcF*, *yibF*, *and yfcG_2*) yielding oxidized glutathione (GSSG) and β-guaiacyl-α-veratryl ethanone (GVE).

We verified two of them did not present the EC number associated with GST activity (EC 2.5.1.18). The remaining sequences presented 98–100% of identity with genes from *Klebsiella* genus (*K*. *Pneumoniae* and *K*. *variicola*) (S2 Table). The closest genes related with the *K*. *variicola* P1CD1 annotated as Glutathione S-transferase (*gst*, *gstB*, *gstB_1*, *gstB_2*, *gstB_3*) and six other genes with EC 2.5.1.18 (Disulfide-bond oxidoreductase–*YfcG*_1 and 2, Glutathionyl-hydroquinone reductase—*yqjG*, Disulfide-bond oxidoreductase—*YghU*, Glutathione S-transferase—*YfcF*, and Putative GST-like protein–*YibF*). We compared the GST found in *K*. *variicola* P1CD1 with the *Lig* genes deposited in the database to identify potentially related functions of the putative GST genes. Considering the identity among *Lig* genes in literature, the analysis showed a reasonable identity but low coverage. The highest coverage (> 50%) was found for the GST of *K*. *variicola P1CD1* assigned as fig|640131.64.peg.784—*yghU*; fig|640131.64.peg.2686—*gstB*_2; fig|640131.64.peg.2808—*gstB*; fig|640131.64.peg.3869 *gstB*_3) with 22.5% of identity with *LigE*, 25.66% with *Lig*G, 25,41% with *LigG*.

**Fig 4:**
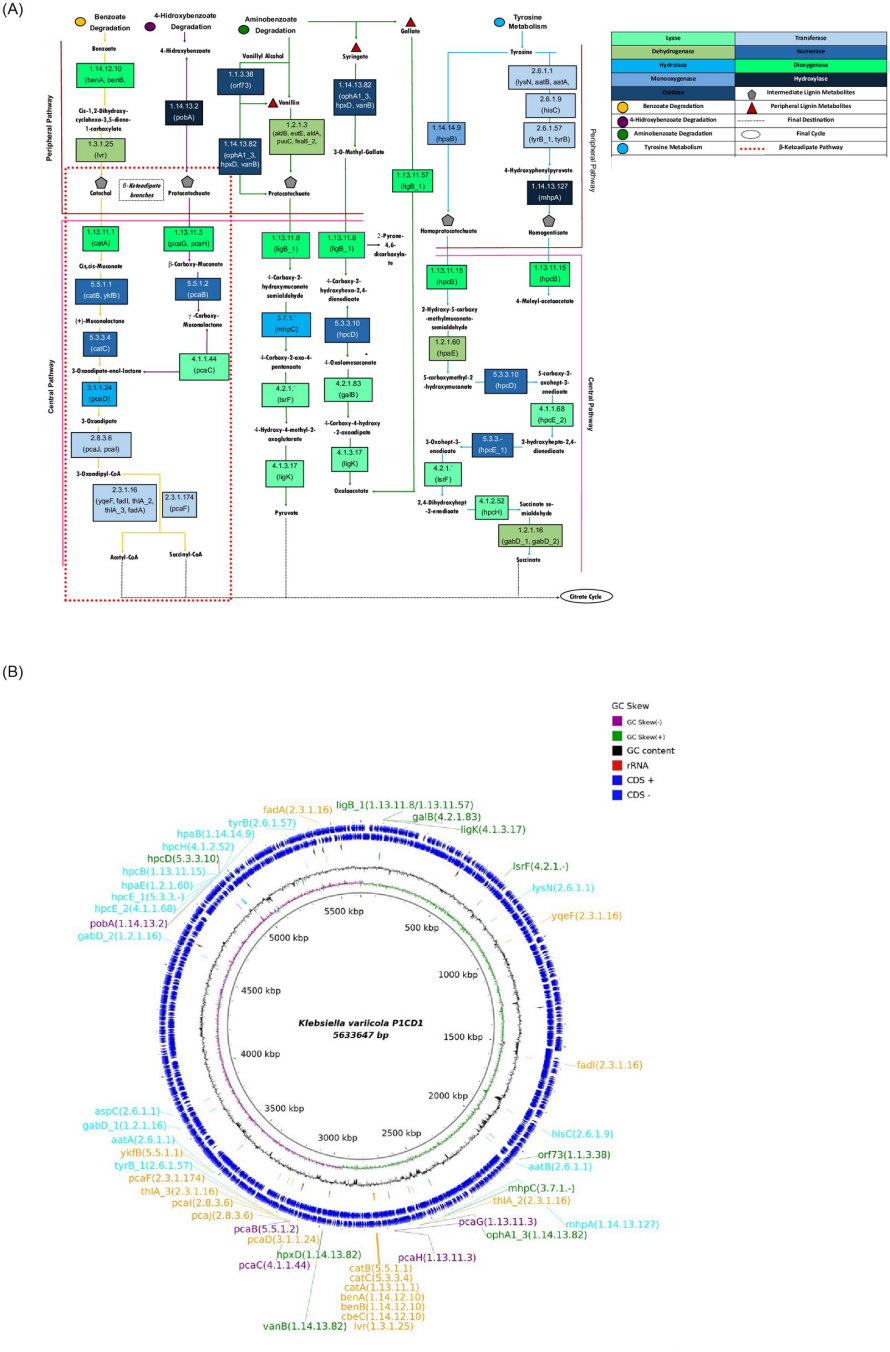
Genome-wide-based metabolic reconstruction for the lignin fragment degradation model in *K*. *variicola* P1CD1. A) The colored circles represent the aromatic compound degradation routes available in the complete second stage of lignin degradation (assimilatory metabolism). The peripheral and central pathways of lignin degradation via β-ketoadipate pathways (via catechol and protocatechuate branches) are highlighted by pink (top) and orange (bottom) lines, respectively. The pentagons represent the central metabolite precursors for deconstruction of ring structures and carbon utilization by cells via acetyl-CoA, pyruvate, and succinate formation. Red triangles represent peripheral intermediates, and gray pentagons represent the central intermediate of lignin degradation. The activities represented in each box are identified according to the color code presented on the right side and based on their respective EC numbers and the name of the genes potentially associated with each metabolic pathway. B) Circular genomic representation and position of the genes present in the lignin fragment degradation model of *K*.*variicola* P1CD1.

*3*.*3*.*3*.*2*. *Stage two (aromatic compounds derived from lignin degradation)*. We identified all the activities needed to perform stage two lignin degradation from the *K*. *variicola* P1CD1 genome, demonstrating its potential to convert the structures resulting from stage one. The repertoire covered all the peripheral and central pathways of aromatic compound degradation metabolism (Fig 4A).

The products are generated due to the peripheral and central metabolic pathways associated with aromatic compound degradation. The catabolic pathways of the aromatic compounds in *K*. *variicola* P1CD1 are part of benzoate degradation, 4-hydroxybenzoate degradation, aminobenzoate degradation, and tyrosine metabolism. These reactions result in the production of lignin intermediates such as catechol, protocatechuate, homoprotocatechuate, and homogentisate. The central pathways are represented by metabolic pathways involved in deconstructing intermediate metabolite chemical rings to generate molecules such as acetyl-CoA, succinyl-CoA, pyruvate, oxaloacetate, and succinate for cellular energy and biomass production. Among the central pathways, we found both β-ketoadipate branches represented by the catechol and protocatechuate in *K*. *variicola* P1CD1, which were associated with the benzoate and 4-hydroxybenzoate metabolisms. According to our model, the conversion of catechol and protocatechuate into acetyl-CoA and/or succinyl-CoA is performed in six main metabolic pathways associated with 17 genes involved in dioxygenase (*catA*, *pcaG*, *and pcaH*), isomerase (c*atB*, *ykfB*, *pcaB*, *and catC*), lyase (*pcaC*), hydrolase (*pcaD*) and transferase activities (*pcaJ*, *pcaI*, *yqeF*, *fadI*, *thlA_2*, *thlA_3*, *fadA*, *and pcaF*).

The metabolic reconstruction suggests that aminobenzoate degradation metabolism plays a pivotal role during lignin degradation in *K*. *variicola* P1CD1 due to its ability to convert vanillin into protocatechuate. Protocatechuate can also be directed to benzoate degradation metabolism via 4-hydroxybenzoate metabolism by its conversion into 3-oxoadipate-enol-lactone. 3-Oxoadipate-enol-lactone represents an intersection between benzoate and 4-hydroxybenzoate degradation metabolism, allowing its conversion to final metabolic products such as acetyl-CoA and succinyl-CoA. On the other hand, protocatechuate also represents an intersection between two metabolisms (4-hydroxybenzoate and aminobenzoate) and can be alternatively converted into pyruvate. Moreover, although the peripheral metabolite syringate is not converted into an intermediate metabolite, *K*. *variicola* P1CD1 aminobenzoate degradation metabolism presents pathways involved in converting syringate into oxalacetate as one of the final products, which may be directed to the citrate cycle in only four steps.

Interestingly, genes identified by the extensive genome-based metabolic reconstruction of benzoate and tyrosine degradation metabolism are present in close genomic contexts (Fig 4B). We observed that the genes involved in both central and peripheral metabolic pathways of benzoate degradation are present in the same genomic context, including the genes involved in converting benzoate into muconolactone (*benA*, *benB*, *cbeC*, *lvr*, *catA*, *catB*, *and catC*). We found other group of genes associated with the peripheral pathways present in the same genomic context and associated with central pathway activities (i.e., *pcaD*, *pcaJ*, *pcaI*, *thlA_3*, *pcaF*, *and yfkB*). The other genes from benzoate degradation metabolism were more distantly arranged (i.e., *yqeF*, *fadA*, *and FadI*) and related to the last steps of this pathway, which may end at acetyl-CoA and succinyl-CoA formation. In tyrosine metabolism, we can observe a group of genes located in a similar genomic context. The dominant group of genes is represented by the activities associated with the peripheral metabolic pathways, mainly those involved with the conversion of homoprotocatechuate and homogentisate into succinate (i.e., *hpcB*, *hpaE*, *hpcE_1 and 2*, *hpcH and gabD_2*). The genes identified for 4-hydroxybenzoate and aminobenzoate degradation metabolism are more widely distributed along the genome.

## 4. Discussion

Bacteria that can depolymerize lignin have already been discovered in a wide range of environments and play an essential role in lignin degradation [25]. Most of the ligninolytic bacterial strains identified belong to Actinomycetes, α-Proteobacteria, and γ-Proteobacteria classes. The genus *Klebsiella* belongs to γ-Proteobacteria, and some members have been successfully identified in microbial consortia associated with the degradation of residual lignin-rich waste from the pulp manufacturing paper industry [26–29]. The successful kraft lignin degradation by the consortia reinforces the importance of syntrophic growth of bacterial members rather than antagonistic effects and the relevance of this interaction to perform lignin deconstruction in natural environments. The contribution of the ligninolytic enzymes from *Klebsiella* in consortia is still unclear but corroborates the importance of bacterial metabolism in environmental lignin mineralization.

The first evidence that *Klebsiell*a members may play an essential role in lignin conversion in the Caatinga biome was obtained from a previous investigation of the microbial community associated with soil and freshwater samples (non-altitudinal region) [30, 31]. The shotgun metagenomic approach revealed that *Klebsiella* sp. represents a minor fraction in the samples from soil and freshwater (< 1%) [30]. However, the predominance (> 90%) of strains belonging to the genus *Klebsiella* (*K*. *oxytoca*, *K*. *pneumoniae*, and *K*. *variicola*) recovered in medium containing lignin as the unique carbon source from the same samples suggests that they can convert lignin in a more efficient way than that of other members of the community [31]. In addition to the phenotypic tests, we obtained the three ligninolytic *Klebsiella draft genomes*, selected due to fast growth in the presence of lignin and dyes as the sole carbon source. Here, we firstly demonstrated the ligninolytic ability of *K*. *variicola* P1CD1 based on phenotypic assays. Initial evidence emerges from it isolating in the presence of lignin as the sole carbon source in cultivated media. However, even well established in identifying ligninolytic strains, there is a criticism about this isolating methods. Most commercial or kraft lignin could present residual organic acids or carbohydrates in your formulation, and it may lead to false negative ligninolytic strains isolation. Thus, we performed a semi-quantitative approach to demonstrate the conversion of nonsoluble lignin by *K*. *variicola* P1CD1. Our results demonstrate a significant fraction of residual nonsoluble lignin was consumed along the bacteria growth. It suggests the *K*. *variicola* P1CD1 convert a fraction of nonsoluble lignin in biomass and promote the release of lignin fragments to the soluble fraction. Interestingly, the ability to grow in the presence of dyes which mimetic lignin fragments reinforce the possibility of *K*. *variicola* P1CD1 to use a part of the solubilized fragments towards biomass formation. Although strong evidence has been found through the presented phenotypic analyzes based on the growth of the *K*. *variicola* P1CD1 strain in the presence of kraft lignin, we have performed genomic analyzes aiming to identify the potential genetic repertoire capable of promoting the ability of the strain to convert lignin and its fragments.

The draft genomes revealed the presence of genes associated with lignin fragment degradation metabolism. Therefore, we hypothesize that ligninolytic *Klebsiella* is ubiquitously distributed in Caatinga and may play essential roles in the soil’s organic carbon cycling. The phenotypic and genomic data from the *K*. *variicola* P1CD1 strain support this hypothesis and suggest that the ligninolytic repertoire involved in lignin fragment degradation is widely distributed among *Klebsiella* strains in Caatinga. Based on the *K*. *variicola* P1CD1 whole-genome sequence, we established a potential ligninolytic genetic repertoire composed of 262 genes distributed among 16 essential activities for lignin degradation. Because the bacterial lignin-degrading systems are still not well established (mainly compared with fungi), we combined different annotation strategies to reconstruct the metabolic pathways involved in lignin degradation. By combining the subsystems, KEGG, and homology-based annotation, we were able to allocate the genetic repertoire according to each metabolic pathway necessary for lignin and aromatic compound degradation available in the databases (RAST, KEGG UniProt, and GenBank). The approach successfully reconstructed the main routes described so far associated with the two stages of lignin degradation. We reconstructed three metabolic routes potentially involving β-aryl ether linkage cleavage associated with phenolic and nonphenolic structures of lignin in the first stage (DyP activity with Mn2+ ion utilization, DyP activity via iron or veratryl alcohol utilization, and laccase activity via Cu1+). We reconstructed the pathways involved with the two branches of the via β-ketoadipate associated with the second stage of lignin degradation, suggesting the ability for dissimilation and assimilation of lignin structures.

Bacterial systems are less oxidatively robust than ligninolytic fungal systems. However, the efficiency of ligninolytic bacterial metabolism emerges from the synergistic attack of the major oxidative enzymes, activating, and uncapping various sites in the lignin molecule [6]. Among the LMEs, we found genes for DyP, multicopper phenol oxidases/laccase, and β-ether/glutathione-S-transferase in the P1CD1 genome. Laccases and DyP are well established as components of prokaryotic lignin-degrading systems [3]. However, laccases can be absent in ligninolytic strains, as observed for *Klebsiella* sp. BRL-6 [8], suggesting a more versatile repertoire involved with lignin degradation in *K*. *variicola* P1CD1.

Laccase (EC 1.10.3.2) is a copper-containing phenoloxidase, which can oxidize electron-rich substrates of phenolic and non-phenolic origin with a concomitant reduction of oxygen to water through a radical-catalyzed reaction mechanism [32]. Although the precise role of laccase enzymes in lignin degradation is uncertain, the deletion of the Streptomyces laccase gene has been shown to lead to reduced amounts of acid-precipitable lignin formation from lignocellulose, implying a role for this laccase in lignin oxidation [33]. Bacterial laccase-like multicopper oxidases have been reported in members of *Bacillus*, *Streptomyces*, *Escherichia*, and *Gramella* bacteria genera [34–41].

Although laccases play a fundamental role in the degradation of lignin, some are directly involved in promoting resistance to copper, which seems to be the case with Cusf found in *K*. *variicola* P1CD1, whose genomic context is surrounded by other genes associated with the resistance process (i.e., efflux pump genes). However, as the role of laccases is quite broad in bacterial metabolism, it may not be possible to rule out some involvement of this group of laccases in the process of lignin degradation. Considering the complex structure of lignin, the use of multiple catalytic strategies and metabolic routes may not be surprising and may offer advantages in different ecological niches.

In contrast, heme peroxidases, such as DyPs, play a central role in the bacterial ligninolytic ability [42]. Heme peroxidases, including DyP (and other lignin peroxidases), are more effective than classical peroxidases for degrading aromatic compounds, constituting 90% of the lignin [43]. The presence of DyPs in ligninolytic bacterial strains appears to be widely distributed in the complete genomes of members belonging to Actinobacteria [44–46, 43] and Proteobacteria [8, 47–49]. The Dyp family promotes the oxidation of Mn(II), and via β-aryl ether lignin model compounds in *R*. *jostii*, RHA1 is one of the best Dyp metabolic roles established thus far [50]. In the *R*. *jostii* RHA1 genome sequence, twenty-six peripheral pathways and eight central pathways are involved in the catabolism of aromatic compounds, including modifications by monooxygenases and dioxygenases. The *Klebsiella* sp. strain BRL6-2 revealed four putative peroxidases, including glutathione and DyP-type peroxidases, and it has a full protocatechuate pathway for catechol degradation into β-ketoadipate, as in *Sphingomonas paucimobilis* SYK6 [8, 47, 51].

The enzyme coded by the *YfeX* gene is a Dyp belonging to the DypB subfamily [50]. Phylogenetic analysis has revealed four discrete Dyp subfamilies identified as A-D, and they may diverge up 85% in sequences within a subfamily [50]. The enzymes characterized to have lignin degradation activity are generally in the DypB and DypC subfamilies. DyP-type peroxidases from *Rhodococcus jostii* RHA1 perform manganese-dependent lignin peroxidase activity, playing a significant role in lignin degradation in this strain [50, 52]. Similarly, the DyPs identified in *Amycolatopsis sp* 75iv2 shows versatile and significant activity for Manganese-peroxidase activity compared to other so far characterized bacterial DyPs. Thus, the presence of the *YfeX* in *K*.*variicola* P1CD1 represents relevant genetic evidence of its ability in degrading lignin. In addition, it indicates a potential degrading ability for phenolic fragments resulting from the degradation process of environmental lignin, which can occur synergistically with other members of the environmental microbial communities, considering the giving the complexity of lignin molecule. Some genes encoding homologs of the *YfeX* have a tight association with genes encoding a bacterial cytoplasmic encapsulating protein referred to as capsulin [53]. Encapsulins form nano compartments that contain peroxidases (and ferritin-like proteins), which are targeted to the interior of encapsulins via unique C-terminal extensions. Encapsulin is involved with enclosing or encapsulating DyPB that possesses a C-terminal signal in their sequences. The *R*. *jostii* RHA1 dypB contains the C-terminal sequence responsible for targeting proteins for encapsulation and is operonically coupled with a sequence 34% identical to encapsulin from *T*. *maritima*, which forms a cellular nano compartment [50]. The genomic context of T. *maritima* encapsulin homologous proteins mostly are part of an operon that codes for encapsulin preceding by a peroxidase [54]. We found a similar genomic context in *K*.*variicola* P1CD1, suggesting an operon structure which may have a functional role in lignin degradation as observed on the mentioned strains.

Moreover, the genome-based metabolic reconstruction revealed that *K*. *variicola* P1CD1 could attack the most abundant bond in the lignin molecule, the β-aryl ether linkage, to metabolize the lignin fragments in the first stage of lignin degradation. Microbial β-etherases belong to the glutathione-S-transferase (GST; EC 2.5.1.18) protein superfamily, which catalyzes the reductive cleavage of β-O-4 bonds. These enzymes are members of the glutathione transferase superfamily (GSTs; EC 2.5.1.18) and cleave the b-aryl ether bond upon glutathione (GSH) consumption. Among a few bacteria well characterize for cleavage of β-O-4 bonds, *Sphingobium* sp. SYK-6 has three different β-etherases (*LigE*, *LigF*, *and LigP*) involved with reductive ether bond cleavage of α-keto-containing β-ether substrates [55–57]. According the postulated catalytic pathway for guaiacyl -alpha-veratrylglycerol (GVL) degradation in S. paucimobilis SYK-6, *LigE*, *LigF*, and *LigP* are b-etherases that cleave the two GVG enantiomers produced, yielding two enantiomeric GSH conjugates (GS-bVG). LigG is the glutathione lyase that uses GSH to liberate veratrylglycerone from GS-b(S)VG through the formation of glutathione disulfide (GSSG). The bacterial β-etherases *LigE* homologous share > 59% amino acid sequence identity with each other and *LigF* homologous share > 39–60% amino acid sequence identity with each other. In contrast, the *LigE* and *LigF* homologous share < 22% amino acid sequence identity between them, forming separated phylogenetic clades with *LigF* homologous [58]. Characterization of heterodimeric beta-etherase from *Novosphingobium aromaticivorans* distinct from *LigF* and *LigE* suggests that the ability to cleave the -aryl ether bond arose independently at least twice in GSTs. *BaeA* and *BaeB* share around 24% amino acid sequence identity and fall into separate phylogenetic clades. *BaeA* is < 36% and < 17% identical to the *LigF* and *LigE* homologues, respectively, and *BaeB* is < 30% and < 18% identical to the *LigF* and *LigE* homologues, respectively [58]. Those findings suggest that the diversity of beta etherase homologous genes may be important for cleaving the variety of aryl ether bonds of lignin-derived oligomers in nature. Considering the identity levels found between the *K*. *variicola* P1CD1 GST genes, *LigE* and *LigG*, we could hypothesize that they may represent a new group of β-etherases involved with lignin degradation, as the *BaeA* and *BaeB* genes, and the activities should be tested and validated biochemically in the near future.

The cleavage of phenolic and nonphenolic structures promotes the formation of peripheral lignin metabolites such as vanillin and syringaldehyde. Peripheral lignin metabolites are converted into intermediates of lignin metabolism, where the molecules can be processed by LDAs. Among the LDAs involved in the second stage of lignin degradation, we found genes for monooxygenases, reductases, hydrolases, dioxygenases, isomerases, and oxidases in P1CD1, which represent activities generally required for the degradation metabolism of aromatic compounds. The ability to degrade lignin provides a link between aromatic degradation and lignin assimilation. Within the ligninolytic genetic repertoire, genes involved in the metabolic pathways for benzoate, 4-hydroxybenzoate, aminobenzoate, and tyrosine production were found, suggesting that the P1CD1 strain can perform assimilation of the lignin fragments originating from the dissimilatory steps in the first stage of lignin degradation.

Here, we explored the KEGG pathways involved in the metabolism of aromatic compounds and found all the genes necessary within the peripheral and central pathways of aromatic compound degradation metabolism. The comparison between the aromatic compound degradation metabolic pathways of *K*. *variicola* P1CD1 and *K*. *variicola* At-22 revealed that although they are strains belonging to the same species, their ligninolytic genetic repertoire populated the metabolic routes established by KEGG differently. The conventional route for the transformation of vanillin into protocatechol is initiated by the catalytic action of vanillin dehydrogenase (EC 1.2.1.67) on vanillate, and the conversion of vanillate to protocatechol is then completed by vanillate O-demethylase (EC 1.14.13.82) [59, 60]. However, P1CD1 uses aldehyde-alcohol dehydrogenase (EC1.2.1.3) to perform direct conversion of vanillin to protocatechol. We identified the potential formation of the intermediates catechol, protocatechuate, homoprotocatechuate, and homogentisate during the peripheral pathways. In most known aromatic-degrading microbes, the upper funneling pathways are linked to the β-ketoadipate pathway by the protocatechuate/catechol catabolic branches [61, 62]. Aromatic degradation has many oxidative pathways involving ortho-cleavage and meta-cleavage of catecholic and protocatechuic molecules to perform ring fission of the intermediates via β-ketoadipate pathways along with the central metabolism of aromatic compounds [63, 64]. The cleavage of these intermediates via β-ketoadipate pathways generates acetyl-CoA, linking catabolism of the more significant aromatic compounds with the TCA cycle. The *K*. *variicola* P1CD1 β-ketoadipate pathways were reconstructed, reinforcing the potential ability of this strain to convert lignin into assimilable fragments, possibly via a protocatechuate/catechol catabolic pathway.

## 5. Conclusion

The combination of phenotypic and genomic assays demonstrated the ligninolytic potential of a newly identified *K*. *variicola* P1CD1 strain isolated from a scarcely characterized Brazilian biome. The genome-wide-based metabolic reconstruction identified metabolic pathways associated with lignin deconstruction via β-aryl ether linkage cleavage and the potential ability to degrade lignin fragments via β-ketoadipate pathways. The metabolic model supports the evidence of possible dissimilatory and assimilatory lignin degradation by *K*. *variicola* P1CD1. Moreover, the complete genome analysis of *K*. *variicola* P1CD1 indicated that in some cases, this bacterium uses alternative routes to the conventional routes described for lignin degradation. However, despite using different routes, the end products still comprise molecules involved in cellular energy and biomass production, such as acetyl-CoA and pyruvate. Thus, the results highlight the ecological relevance of members from the environmental *Klebsiella* genus in lignin degradation and open the possibility to explore its ligninolytic genetic repertoire for biotechnological applications involving the valorization of coproducts from lignin deconstruction.

## Supporting information

S1 Fig Growth in the presence of kraft lignin. A) Solid media in 24 hours and B) Growth curve in liquid media revelead a exponential growth between 12 and 96 hours.
(PDF)

S2 Fig Circular map view of the whole-genome reconstruction of ligninolytic *Klebsiella variicola* P1CD1 isolated from altitudinal Caatinga sediments. From outside to center, rings 1 and 2 show protein-coding genes oriented in the forward and reverse directions; ring 3 shows RNA sequences in red arrows; ring 4 shows the G + C% content plot; and the innermost ring shows the GC skew, with purple indicating negative values and green indicating positive values.
(PDF)

S3 Fig Classification of proteins in the subsystem category distribution identified in *Klebsiella variicola* P1CD1. (PDF)

S4 Fig The ligninolytic genetic repertoire found in the *K*. *variicola* P1CD1 complete genome. The genes were identified based on the activities associated with lignin (and fragments) and aromatic compound degradation metabolism established by RAST metabolic models and the literature.
(PDF)

S5 Fig The genetic repertoire of *Klebsiella variicola* P1CD1 for lignin and aromatic compound degradation (distribution of the enzymes present in degradation subsystems of lignin and aromatic compounds). (PDF)

S6 Fig The genetic repertoire of *Klebsiella variicola P1CD1* for lignin and aromatic compound degradation (clustering of the enzymes not present in degradation subsystems of lignin and aromatic compounds—out of subsystems). (PDF)

S1 Table List of the genetic repertoire associated with lignin and fragment lignin degradation found in the *Klebsiella variicola* P1CD1 complete genome, found in and out of subsystems. (XLSX)

S2 Table List of genes identified in the genome-wide-based metabolic reconstruction of stage one and two lignan degradation in *Klebsiella variicola* P1CD1. (XLSX)
