# Supplementary figures and images for: Complete genome reveals genetic repertoire and potential metabolic strategies involved in lignin degradation by environmental ligninolytic Klebsiella variicola P1CD1
Source: PLoS One. 2020 Dec 22;15(12):e0243739. doi: 10.1371/journal.pone.0243739 (PMC7755216; doi:10.1371/journal.pone.0243739)

## Growth in kraft lignin

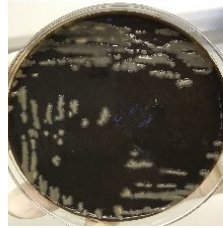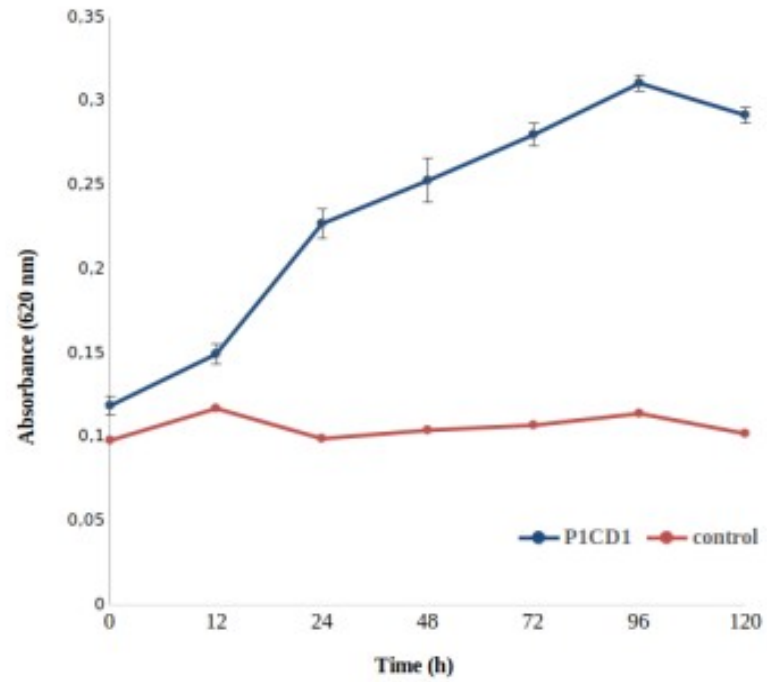

Supplement: S1 Fig — A) Solid media in 24 hours and B) Growth curve in liquid media revelead a exponential growth between 12 and 96 hours. (PDF) [file pone.0243739.s001.pdf]

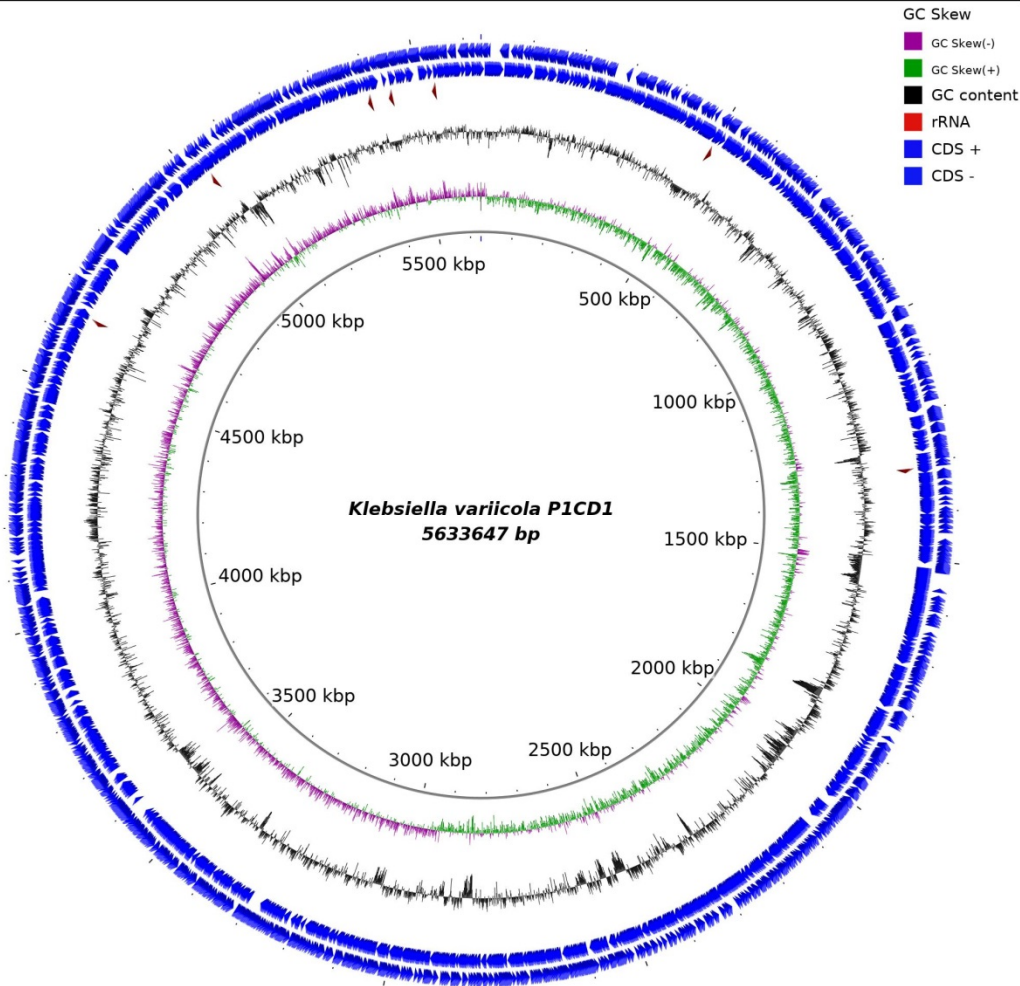

Supplement: S2 Fig — From outside to center, rings 1 and 2 show protein-coding genes oriented in the forward and reverse directions; ring 3 shows RNA sequences in red arrows; ring 4 shows the G + C% content plot; and the innermost ring shows the GC skew, with purple indicating negative values and green indicating positive values. (PDF) [file pone.0243739.s002.pdf]

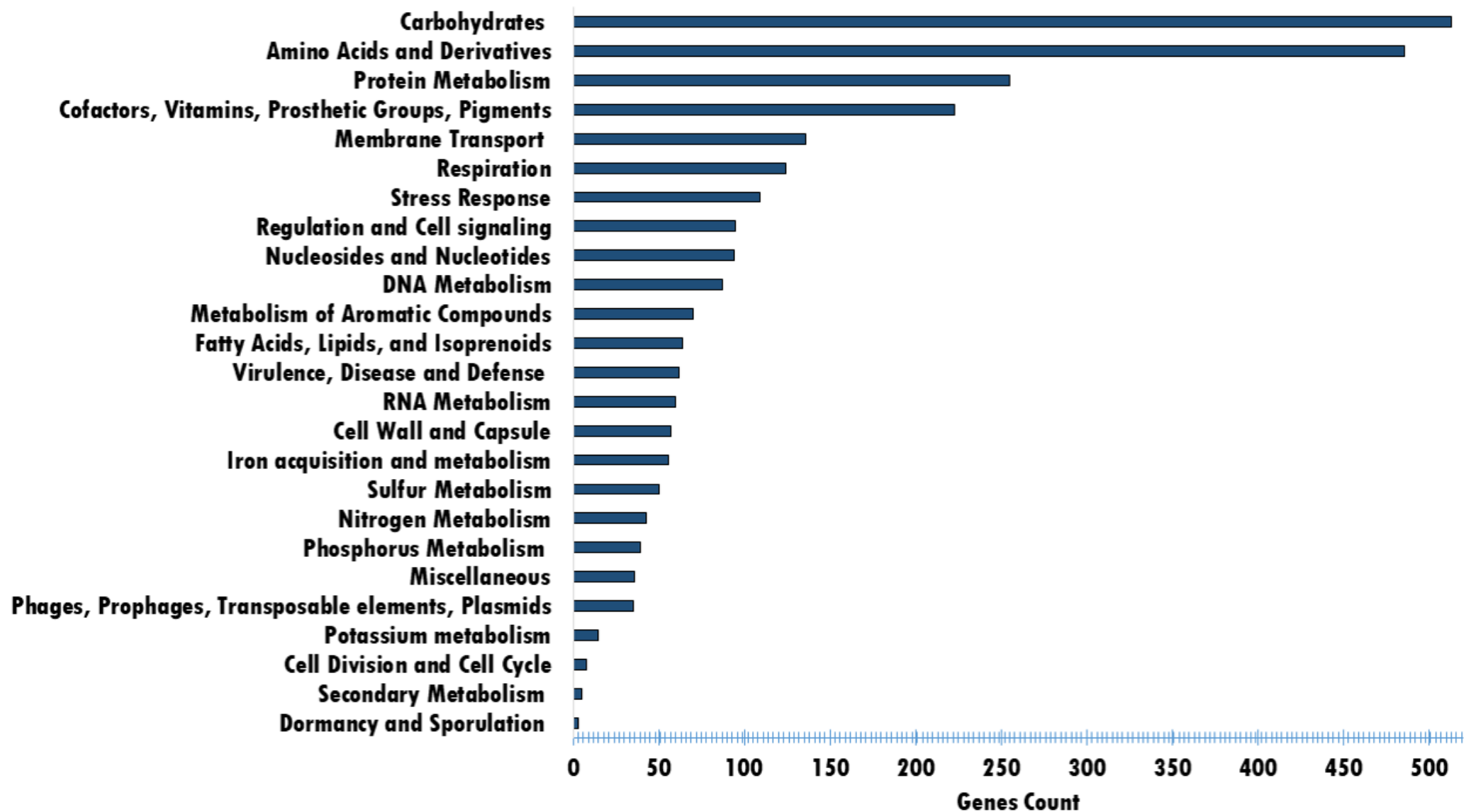

Supplement: S3 Fig — (PDF) [file pone.0243739.s003.pdf]

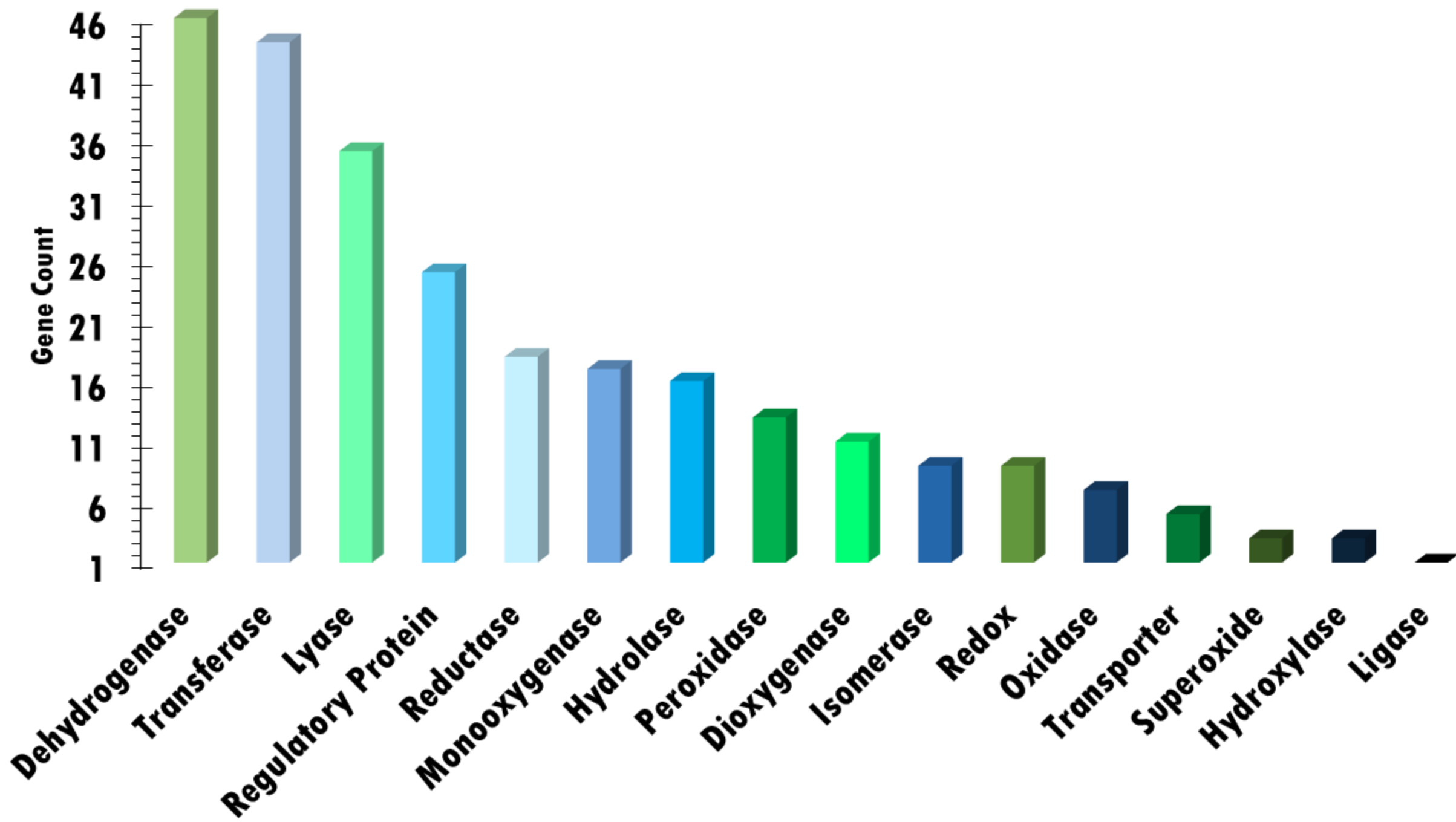

Supplement: S4 Fig — The genes were identified based on the activities associated with lignin (and fragments) and aromatic compound degradation metabolism established by RAST metabolic models and the literature. (PDF) [file pone.0243739.s004.pdf]

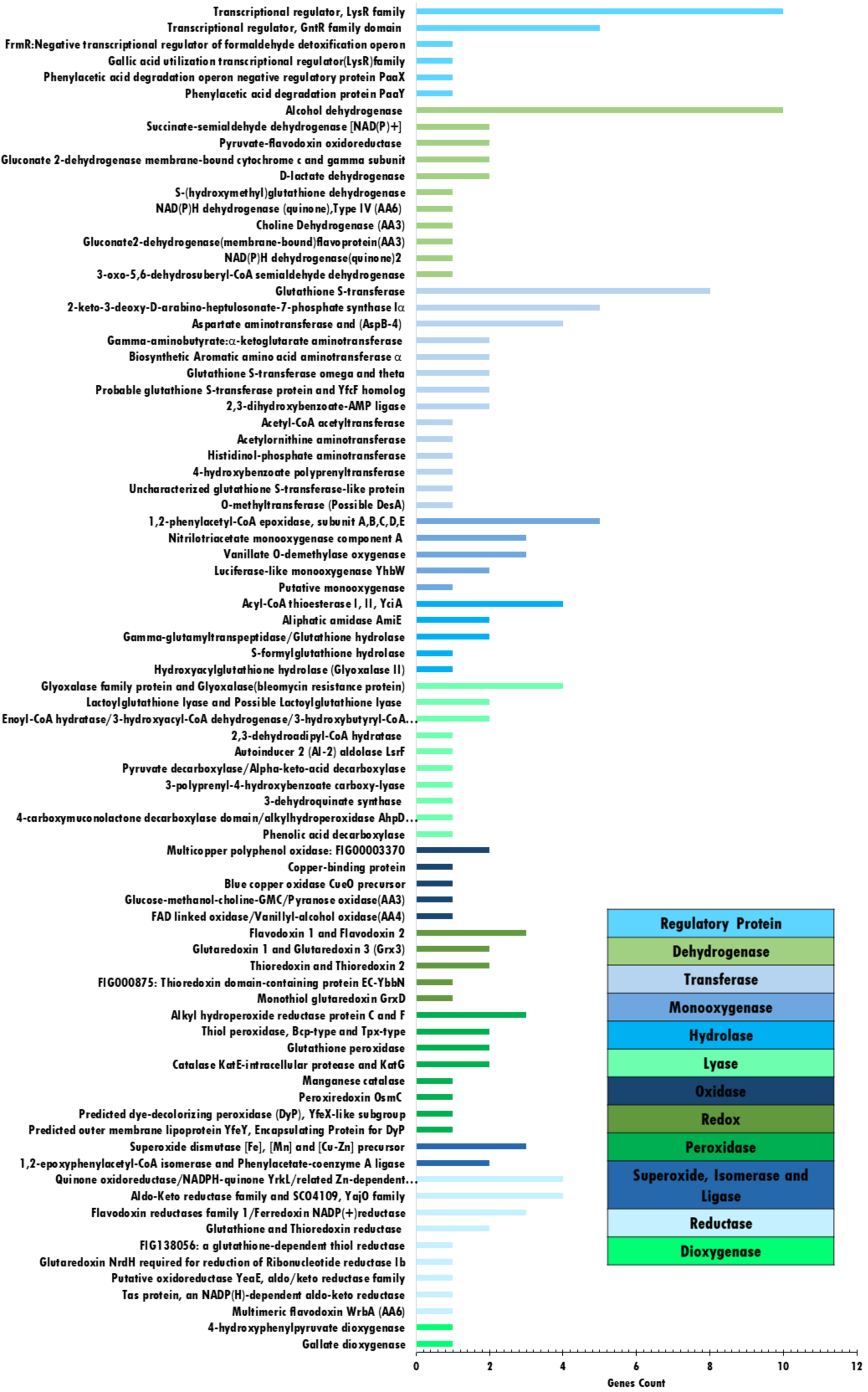

Supplement: S5 Fig — (PDF) [file pone.0243739.s005.pdf]

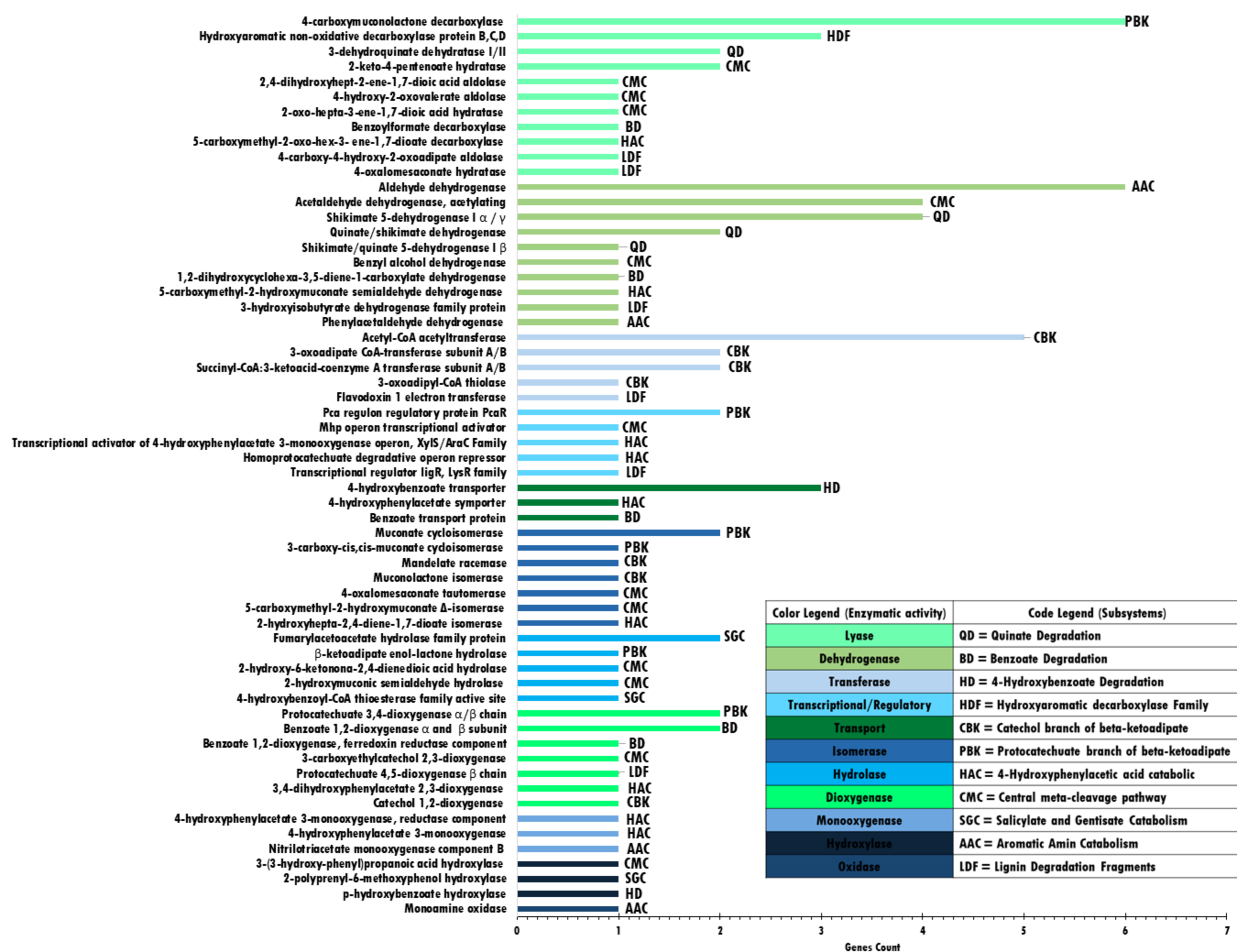

Supplement: S6 Fig — (PDF) [file pone.0243739.s006.pdf]
